# Mr. Harrup on Tinea Capitis

**Published:** 1811-09

**Authors:** Robert Harrup

**Affiliations:** Chobham


*To the Editors of the Medical and Physical Journal.*

### *Mr. Harrup on Tinea Capitis.*

GENTLEMEN,

HAVING of late years met with a considerable number of cases of Tinea Capitis and Ring-worm, I take the liberty of sending you the result of my experience in answer to the note in your last number signed *Philanthropos.*

As the remedies I have employed have proved uniformly successful when duly persevered in, and been used indiscriminately in both diseases, I shall only state a few cases by way of illustration.

In March, 1807, two most inveterate cases of Tinea Capitis in the same family came under my care—the one, a girl of eight, the other, a weak, unhealthy boy about six years of age. The disease had continued upwards of two years, and various remedies had been employed by both regular practitioners and empirics, but without success. Although their heads had been formerly frequently shaved, it was now impossible to apply the razor; the hair was therefore cut as close as possible, and after well washing the parts affected with warm water and soft soap, a lotion composed of two drachms of sulphurated kali, and eight ounces of lime-water was applied night and morning. They also took from eight to ten grains of the hydrarg. cum sulph. twice a day; these remedies were persevered in about three weeks, with two or three doses of calomel and rhubarb in that time, when the parts had so much recovered as to bear shaving. The hydrarg. cum sulphure was continued as usual, and the following liniment, recommended commended by Mr. Earnest in the 17th volume of this Journal, was substituted for the lotion. R Sapon. mollis ℥iss, potassæ sulphuret. pulv. piper. nig. āā ʒiij. M. ft. liniment. After continuing this plan two months longer the boy's head was quite well, and the girl's exhibited only some slight streaks of scurf, which was soon removed by the daily application of a small quantity of the ung. hydrarg. nitric. oxyd. Oil-skin caps, fitting close to the head, were worn during the whole time of the cure. Not the smallest trace of the disease has appeared upon either of them since.

In July 1808, a nobleman's son, about fourteen years of age, was placed under my care for ring-worm of the head, which had subsisted some time. At the request of a near relation, two different applications, said to be efficacious in such cases, were tried, but with no good effect. The above mentioned liniment was then had recourse to and applied twice a day; he also took a scruple of the hydrarg. cum sulph. daily in the form of an electuary In 24 hours the parts were nearly well, and the cure was completed in about six days longer, by the application of the ung. hydrarg. nitric. oxyd. No return of the disease since

In the latter end of 1809, a child four years old, who laboured under a similar complaint, which had subsisted a considerable time, and for which a great variety of medicines had been unsuccessfully employed, was completely cured in the course of two months by the same means.

Ring-worm made its appearance in a school in this neighbourhood the beginning of last year; several boys were infected, and the school in consequence broke up. Four that remained were put under my care. After some delay their heads were shaved, and oil-skin caps provided. The cure was begun by a dose of calomel and rhubarb, and the hydrarg. cum sulph. from six grains to twelve, were administered twice a day and persevered in. To the parts affected, and indeed over the whole of the head, the following lotion, which is recommended by Mr. Barlow in the 14th volume of this Journal, was applied every night and morning, the parts being previously washed with warm water and soft soap. R. Potassæ sulphuret. (recens pp.) ʒiij. sapon. alb. Hyspan. ʒiss. aq. calcis ℥viiss. spir. vinos. rect. ℥ss. M. ft. lotio. In the course of two months they were so much recovered that the lotion was discontinued, and the cure completed by daily rubbing the parts with a small quantity of the ung. hydrarg. nitrat.

The disease has not returned. I consider the exhibition of the hydrarg. cum sulph. as greatly contributing to the cure of these obstinate disorders. In some cases I have tried to effect a cure a cure by the external applications alone, but the progress was so slow that I was ultimately under the necessity of having recourse to the hydrarg. cum sulph.; of late I have generally administered it conjoined with a few grains of magnesia or kali nitrat. and in somewhat larger doses than what is usually prescribed.

If the statement of the foregoing cases is deemed sufficient to induce Philanthropos, and others of your numerous readers, to make trial of the means my experience warrants me to recommend; the result, I hope, will be published through the medium of your Journal; and if success be attained, much satisfaction will be afforded to,

Gentlemen,

Your obedient humble Servant,

ROBERT HARRUP.

